# The process of hypertension induced by high-salt diet: Association with interactions between intestinal mucosal microbiota, and chronic low-grade inflammation, end-organ damage

**DOI:** 10.3389/fmicb.2023.1123843

**Published:** 2023-02-28

**Authors:** Tao Zheng, Yi Wu, Kang-xiao Guo, Zhou-jin Tan, Tao Yang

**Affiliations:** ^1^College of Food Science and Engineering, Central South University of Forestry and Technology, Changsha, China; ^2^School of Pharmacy, Hunan University of Chinese Medicine, Changsha, China; ^3^Medical School, Hunan University of Chinese Medicine, Changsha, China

**Keywords:** high-salt diet, hypertension, inflammation, end-organ damage, intestinal mucosal microbiota

## Abstract

Inflammation and immunity play a major role in the development of hypertension, and a potential correlation between host mucosal immunity and inflammatory response regulation. We explored the changes of intestinal mucosal microbiota in hypertensive rats induced by high-salt diet and the potential link between the intestinal mucosal microbiota and inflammation in rats. Therefore, we used PacBio (Pacific Bioscience) SMRT sequencing technology to determine the structure of intestinal mucosal microbiota, used enzyme-linked immunosorbent assay (ELISA) to determined the proinflammatory cytokines and hormones associated with hypertension in serum, and used histopathology methods to observe the kidney and vascular structure. We performed a potential association analysis between intestinal mucosal characteristic bacteria and significantly different blood cytokines in hypertensive rats induced by high-salt. The results showed that the kidney and vascular structures of hypertensive rats induced by high salt were damaged, the serum concentration of necrosis factor-α (TNF-α), angiotensin II (AngII), interleukin-6 (IL-6), and interleukin-8 (IL-8) were significantly increased (*p* < 0.05), and the coefficient of immune organ spleen was significantly changed (*p* < 0.05), but there was no significant change in serum lipids (*p* > 0.05). From the perspective of gut microbiota, high-salt diet leads to significant changes in intestinal mucosal microbiota. *Bifidobacterium animalis* subsp. and *Brachybacterium paraconglomeratum* were the dominant differential bacteria in intestinal mucosal, with the AUC (area under curve) value of *Bifidobacterium animalis* subsp. and *Brachybacterium paraconglomeratum* were 1 and 0.875 according to ROC (receiver operating characteristic) analysis. Correlation analysis showed that *Bifidobacterium animalis* subsp. was correlated with IL-6, IL-8, TNF-α, and Ang II. Based on our results, we can speculated that high salt diet mediated chronic low-grade inflammation through inhibited the growth of *Bifidobacterium animalis* subsp. in intestinal mucosa and caused end-organ damage, which leads to hypertension.

## 1. Introduction

Hypertension is a major public health problem affecting people all over the world (Mills et al., 2020). In China, more than 270 million people suffer from hypertension, which making it became one of the most common chronic diseases (The National Bureau of Disease Control and Prevention et al., 2020). Elevated blood pressure is one of the leading causes of cardiovascular disease, resulting in more than 10 million deaths in 2019 (Bundy et al., 2017). Unhealthy diet (high sodium, low potassium, alcohol consumption), obesity and physical inactivity were major risk factors for hypertension (Wu et al., 2022). Dietary intervention was closely related to human health and disease. The World Health Organization (WHO) recommends no more than 5 g of salt per day for adults and even less for children, but because of dietary habits and the food industry, people’s daily salt intake far exceeds WHO recommendations (World Health Organization, 2012; Tan et al., 2019). Because excessive salt intake is considered a major cause of high blood pressure and other cardiovascular diseases, public health guidelines in many countries widely recommend that people reduce their dietary salt intake (Whelton et al., 2018; Williams et al., 2018; Writing Group of 2018 Chinese Guidelines for the Management of Hypertension, 2019). Dietary salt reduction is one of the most effective measures to prevent blood pressure, and the American Heart Association affirmed high salt intake as an independent cardiovascular risk factor (Elijovich et al., 2016). This paper focuses on the study of hypertension induced by high salt intake.

Hypertension is considered a low-grade inflammation characterized by increased levels of various pro-inflammatory cytokines (Mehaffey and Majid, 2017). Immunity and inflammation of hypertension involve numerous cell types and secreted factors in complex physiological and pathological processes (Van Beusecum et al., 2022). From the fact that salt can directly regulate the phenotypes of T cells and myeloid cells, we can see the bidirectional mechanism of sodium and immune system in regulating blood pressure. There was more and more suggestions that the immune system played an important role in sodium homeostasis, for example, studies of inflammatory cells and their secreted effector have provided important insights into salt sensitivity (Rucker et al., 2018). The level of inflammatory biomarkers (high-sensitive C-reactive protein and various cytokines) were increased in patients with hypertension and the production of these biomarkers involves different cells, for example, interleukin-1β (IL-1β) is produced by myeloid cells, and interleukin-1α (IL-1α) is released by epithelial cells and endothelial cells (Xiao and Harrison, 2020). Pro-inflammatory T cell aggravate hypertension by releasing pro-inflammatory cytokines, such as interleukin-17A (IL-17A) and TNF-α (Li et al., 2023). With the development of microbiology and many scientific technologies, the complex interaction of food, microorganisms and their metabolites with the intestinal cavity and intestinal mucosa and the immune system has been revealed (Clemente et al., 2018).

The intestinal mucosa is the largest immune organ and it separated the intestinal cavity from the lamina propria (Santisteban et al., 2017). The lamina propria contains immune cells and the epithelium could maximize the absorption of nutrients while preventing the passage of bacteria and food (Bron et al., 2011). Hypertension is associated with increased intestinal permeability and induces a pro-inflammatory response in the host (Chen et al., 2020). A large number of microbial species were attached to the intestinal mucosa of mammals, which were different from those in the intestinal contents (Schären and Hapfelmeier, 2021). Human intestinal microbiota were related to metabolism, immunity and inflammation, which provided a new insight for the study of hypertension pathogenesis (Liu et al., 2020). Hypertensive patients and animal models showed dysregulation of gut microbiota which increased the risk of hypertension (Tang et al., 2019). The intestinal mucosa is the first space for the interaction between gut microbiota and the host, suggesting the attention demand to the contribution of intestinal mucosal microbiota to the hypertension induced by high salt diet. Genomics offered the novel opportunities to identify and characterize bacterial effector molecules that cause intestinal mucosal responses. Adaptive and innate immune systems and low-grade inflammation may play an important role in the development and progression of hypertension (Formanowicz et al., 2020). Although there are many studies on hypertension, most of them focus on the correlation between changes in intestinal microbiota and intestinal metabolites and hypertension. There were few studies on the role of bacteria attached to intestinal mucosa in mucosal immune function of high salt-induced hypertension. The composition of feces, intestinal microflora and intestinal mucosal microbiota was significantly different, and the intestinal microbiota is mainly responsible for the metabolism of food and drugs, while the intestinal mucosal microbiota is more related to the immunity and disease (Tropini et al., 2017). Compared with the gut microbiota, intestinal mucosal microbiota were more stable, and changes in their diversity, structure and function could affect the immune response of the organism. In-depth study of intestinal mucosal microbiota characteristics of hypertensive was a very important part to study the pathogenesis of hypertension from the perspective of intestinal microecology. Based on the above, this study focused on the relationship between intestinal mucosal microbiota and hypertension induced by high-salt diet. In this study, we measured the changes in blood indicators associated with hypertension, explored the characteristics of gut mucosal miocrobiota and did the correlation analysis between blood indicators and differential bacteria. We hope that our research could provide a basis for exploring the prevention and treatment of high salt diet-induced hypertension from the perspective of inflammation and intestinal mucosal immunity.

## 2. Materials and methods

### 2.1. Animals

Male Wistar rats (4 weeks old, 75–95 g) were purchased from Beijing Vital River Laboratory Animal Technology Co., Ltd. (Beijing, China), with license number SCXK (Jing) 2016–0006. Wistar rats were raised in a shielded environment at the Animal Experiment Center of the Hunan University of Chinese Medicine with license number SYXK (Xiang) 2015–0003 under a 12/12 dark–light cycle (21 ± 2°C with a relatively constant humidity of 45 ± 10%). Rats were provided *ad libitum* access to food and water.

### 2.2. Hypertension modeling induced by a high salt diet

Twelve Wistar rats were randomly divided into a normal group (MZ group) and hypertensive group (MG group) (six rats per group). Rats in the MG group were given 8% high salt animal feed and those in the MZ group were given normal animal feed. High salt feed and normal feed were obtained from the same feed company (Beijing Vital River Laboratory Animal Technology Co., Ltd.) and had the same nutrient contents, except for the salt content. In the analysis of the development of hypertension, blood pressure was measured by rat caudal artery manometry once a week. After 6  weeks, the rats developed stable hypertension.

### 2.3. Hypertension related blood indicators and lipid profile analysis

The rats fasted for 12 h before sampling, and after the rats were sacrificed, blood was collected into the procoagulant tube. The whole blood of rat was centrifuged at 3000 R/min for 10 min, and the serum was taken for standby. The concentration of IL-6, IL-8, IL-1β, TNF-α, norepinephrine (NE), AngII and leptin (LEP) were determined by ELISA. ELISA Kit was provided by Jiangsu Feiya Biotechnology Co., Ltd. Blood lipids in serum samples were determined by the automatic biochemical instrument, such as serum total cholesterol (TC), triacylglycerol (TG), high-density leptin cholesterol (HDL-C), and low-density leptin cholesterol (LDL-C).

### 2.4. Organ collection and calculation

After the rats were sacrificed under anesthesia, the organs (liver, spleen, and thymus) were quickly dissected and removed, the fat and fascia were removed, and the surface liquid was blotted dry with filter paper. Organ index was calculated as organ weight divided by body weight of rats.

### 2.5. Pathological slides of blood vessel

Rats were euthanized, and blood vessels and kidneys samples were collected. Immediately upon removal, blood vessels and kidneys samples were immersion fixed in 10% buffered formalin. The formalin-embedded tissues were gradually dehydrated, embedded in paraffin, cut into 5 μm sections, deparaffinized, and eventually stained using the hematoxylin and eosin (H&E) method. The histology of H&E-stained sections was checked out by an expert pathologist, using light microscopy supplied with a digital camera under a magnification of 100x and 200x.

### 2.6. Intestinal mucosa collection

The intestinal tract tissue from the pylorus of the stomach to the ileocecus was cut longitudinally with sterile scissors to peel off the contents. The mucosa of intestinal tract tissue stripped of contents was scraped with a slide (Xie et al., 2019). Intestinal mucosa samples in the MZ group and MG group were collected in sterilized tubes and stored in a −80°C freezer for 16S rRNA PacBio SMRT gene full-length sequencing.

### 2.7. 16S rRNA gene full-length sequencing

PacBio SMRT sequencing technology was used to accurately obtain full-length 16S rRNA gene sequences (Chen et al., 2020; Escapa et al., 2020). Total microbial genomic DNAs of intestinal mucosa samples were extracted following the manufacturer’s instructions and stored at-20°C. Total microbial genomic DNA samples were extracted using the OMEGA DNA Isolation Kit (D5625-01; Omega, Knoxville, TN, United States) following the manufacturer’s instructions. The DNA concentration was determined using the NanoDrop ND-1000 spectrophotometer (Thermo Fisher Scientific, Waltham, MA, United States). PCR amplification of the nearly full-length bacterial 16S rRNA gene was performed using the forward primer 27F (5′-AGAGTTTGATCMTGGCTCAG-3′) and the reverse primer 1492R (5′-ACCTTGTTACGACTT-3′). The extracted DNA was amplified by two-step PCR, with sample-specific 16 bp barcodes incorporated into the forward and reverse primers for multiplex sequencing in the second PCR step. Next, the TruSeq Nano DNA LT Library Prep Kit was used to prepare the sequencing library. The library was tested using the Agilent High Sensitivity DNA Kit on the Agilent Bioanalyzer (Santa Clara, CA, United States). Finally, the amplified DNA fragment was sequenced using the MiSeq sequencer to obtain 2 × 300 bp paired-end reads with the MiSeq Reagent Kit V3 (600 cycles). SMRT sequencing technology and the PacBio Sequel platform were used for analyses at Shanghai Personal Biotechnology Co., Ltd. (Shanghai, China).

### 2.8. Bioinformatics and statistical analysis

Gut mucosal microbiota by high throughput sequencing of 16S rRNA were analysized, and sequences with similarity higher than 97% are assigned to an OTU (Wang et al., 2018). Chao1 and ACE indexes reflect the abundance of the community, and the larger the index, the higher the abundance of the community. Simpson and Shannon indexes reflect the community diversity, and higher index values indicate higher community diversity. The beta diversity analysis examine the similarity of community structure among different samples. The three main methods, principal coordinate analysis (PCoA), non-metric multidimensional scaling (NMDS) and clustering analysis, are used to naturally decompose the community data structure and rank the samples by Ordination to observe the differences between samples (Bray and Curtis, 1957). LEfSe (Linear discriminant analysis Effect Size) and random forest analysis detected groups that differ significantly in the abundance of gut mucosa and identifies potential biomarkers (Breiman, 2001; Edgar, 2013). The receiver ROC was plotted and the AUC was calculated to analyze the value of differential flora in predicting disease. Canonical correspondence analysis (CCA) and Correlation heatmap was used to investigate the association of blood indicators and gut mucosal microbiota. GraphPad Prism (version 7.04) and IBM SPSS Statistics for Windows, version 20 (IBM Corp., Armonk, NY, United States) were used for statistical analysis. The results were analyzed using the one-way ANOVA and Bonferroni *post hoc* multiple comparison test to evaluate the significance of the differences between the animal groups. Results with *p* < 0.05 were considered as statistically significant.

## 3. Results

### 3.1. High-salt diet induced hypertension in rats, leading to behavior and organ coefficient changed

**Figure 1:**
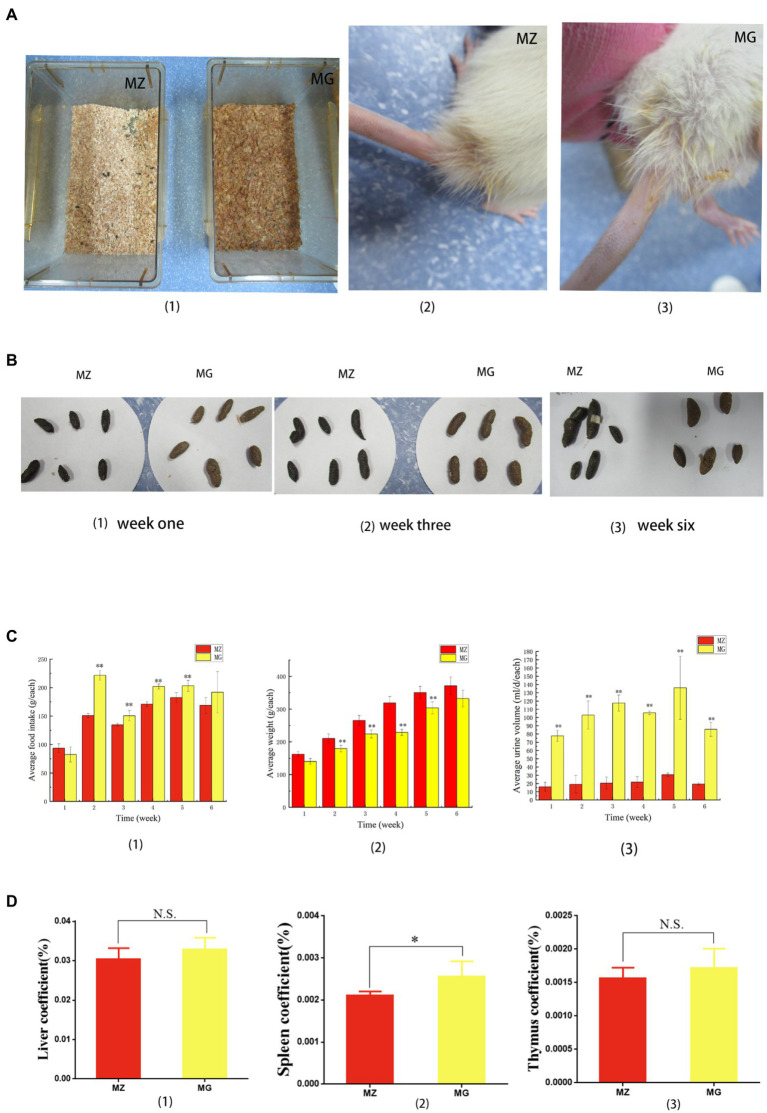
High-salt diet induced behavioral and organ coefficient changes in rats. **(A)** (1) Sawdust bedding material of rats in MZ group and MG group, (2, 3) showed the tail of rats in MZ group and MG group, respectively. **(B)** Feces of rats in MZ and MG groups at week one (1), week three (2), and week six (3). **(C)** Changes in food intake (1), body weight (2), and urine output (3) during hypertension induced by high-salt diet in MZ and MG groups. **(D)** Liver coefficient (1), spleen coefficient (2) and kidney coefficient (3) of rats in MZ and MG groups. MZ, normal group, MG, hypertensive group. **(A–D)** *p < 0.05, **p < 0.01; N.S., not significant (*p* > 0.05).

Food intake, urine output, defecation, body weight, and mental status of rats were significantly altered during the period of hypertension induced by high-salt diet. The rats in MZ group had normal mental state, normal diet, normal defecation and urine output, and smooth fur (Figures 1A1–3,B1–3,C1–3). It can be seen from the feces of rats in the first, third and sixth weeks that the color of feces of rats eating high-salt diet was different from that of rats eating normal diet. In the third and sixth weeks that the feces of MZ group was smooth and the feces of the MG group was dry, which suggested that the rats on the high-salt diet may have constipation (Figures 1B1–3). The food intake of MG group was significantly higher than that of the MZ group from the second to the fifth week (*p* < 0.05), but there was no significant difference between the two groups for the sixth week (*p* > 0.05) (Figure 1C1). From the second week, the body weight of the MZ group was significantly higher than that of the MG group (*p* < 0.05), and there was no significant difference at the sixth week (*p* > 0.05) (Figure 1C2). Animals were placed in a metabolic cage and urine was collected for 24 h, and the high-salt diet resulted in a significant increase in urine output. The changes of the urine output also indicated by the sawdust bedding material and the wetness of the tail fur of the rats (Figures 1A1–3). According to the metabolic experiment of rats, from the first week of high-salt diet intake, the urine output of hypertensive rats was significantly higher than that of MZ group (*p* < 0.05), and the urine output of hypertensive rats was about 4–6 times that of normal rats (Figure 1C3). Compared with the MZ group, the rats in MG group were hyperactive and exercised more, the rats developed stable hypertension from the fifth week.

**Figure 2:**
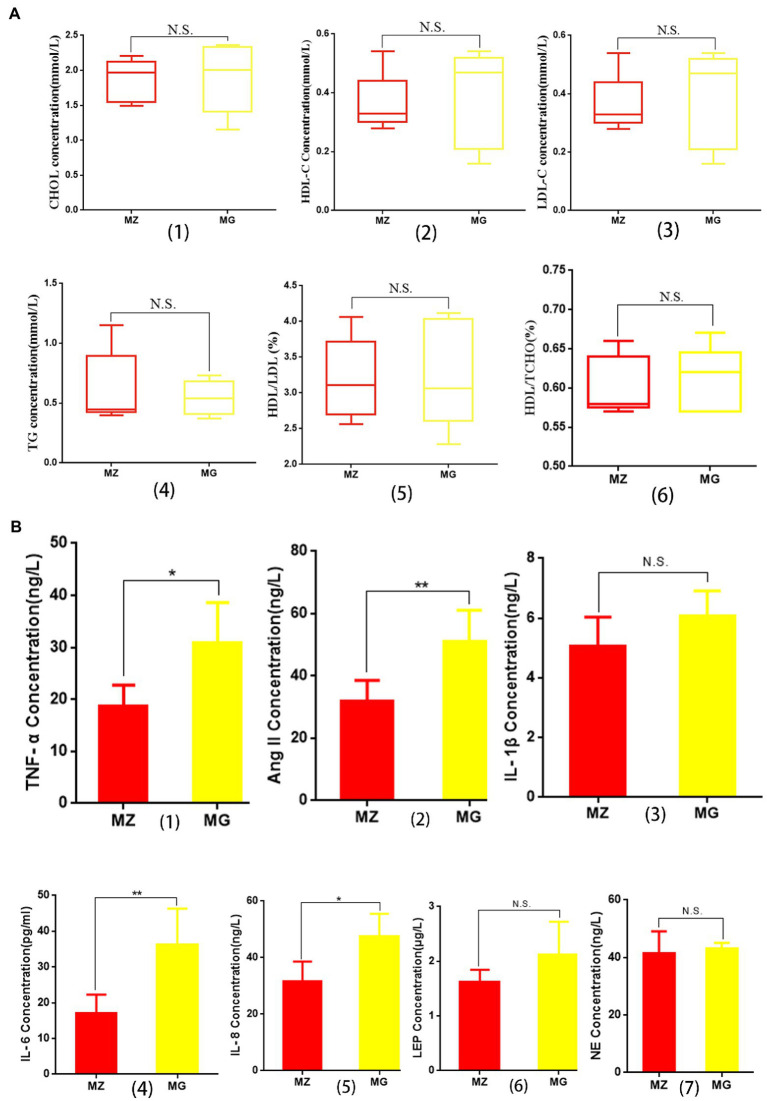
Effects of high salt diet on blood lipids and inflammatory cytokines and hormones related to hypertension in rats. **(A)** Changes of blood lipid in MZ group and MG group. **(B)** Changes in inflammatory cytokines and hormones associated with hypertension in MZ and MG groups. MZ, normal group; MG, hypertensive group.

**Figure 3:**
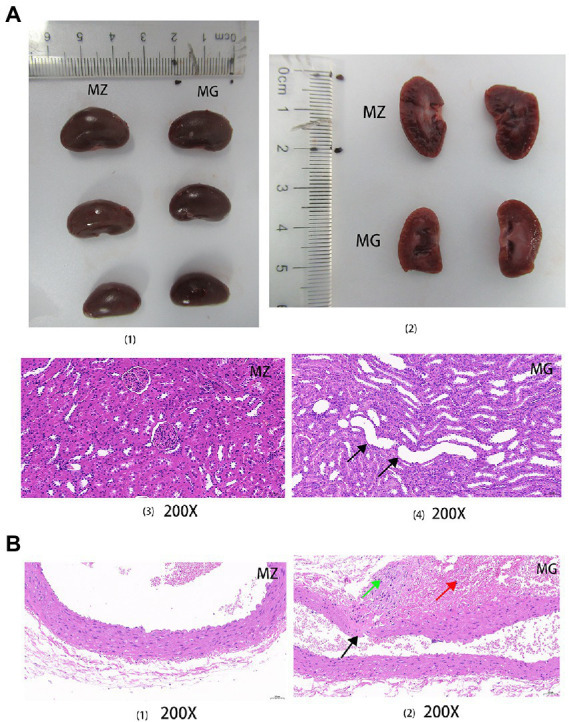
High salt diet caused kidney and blood vessel damage in rats. **(A)** Complete and sectional views of the kidneys in the MZ and MG groups (1, 2); HE staining sections of kidney in the MZ and MG groups (3, 4). **(B)** HE staining sections of vascular in the MZ and MG groups. MZ, normal group, MG, hypertensive group. Solid arrows of different colors indicated damage to the organs.

In terms of organ coefficient, the liver coefficient and thymus coefficient of MZ group and MG group were 0.304, 0.329 and 0.016, 0.017, respectively, and there was no significant difference between the two groups (*p* > 0.05) (Figures 1D1). The spleen coefficient was 0.021 and 0.025, respectively, and spleen coefficient between the two groups was significant difference (*p* < 0.05) (Figure 1D2).

### 3.2. High salt diet leads to change in blood indexes and organ damage related to hypertension in rats

#### 3.2.1. High salt diet-induced hypertension has no effect on blood lipids

The concentrations of HDL-C, LDL-C, and CHOL in serum of the hypertensive rats were higher than MZ group, and the concentration of TG was lower than MZ group, but there was no significant difference in the four indexes between the two groups (*p* > 0.05) (Figures 2A1–4). There were no significant differences in the ratios of HDL/TCHO and HDL/LDL between the two groups (Figures 2A5,6). The results indicating that although high salt diet caused hypertension in rats, it did not significantly change the level of blood lipid and the value of HDL/LDL and HDL/TCHO.

**Figure 4:**
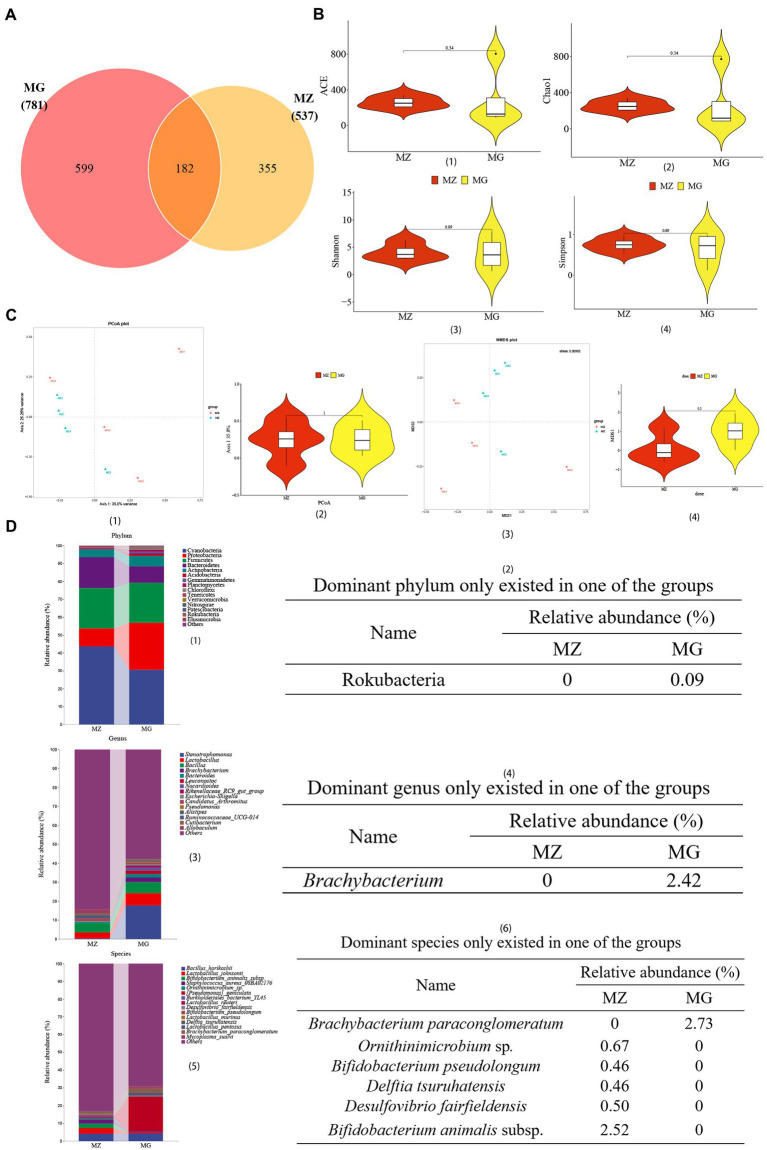
High-salt diet induced changes in OTU annotation, gene richness, and microbial composition in rats. **(A)** Venn diagram of OTU. **(B)** Distribution illustration of samples gene representative of OTU number in community (ace index) (1), community richness (chao1 index) (2), and community diversity (Shannon index and Simpson index) (3, 4). **(C)** PCoA plot (1, 2), NMDS plot (3, 4). **(D)** Dominant intestinal mucosal microbiota composition and dominant microbiota only existed in one of the groups at the level of phylum (1, 2), genus (3, 4), and species (5, 6). MZ, normal group, MG, hypertensive group.

**Figure 5:**
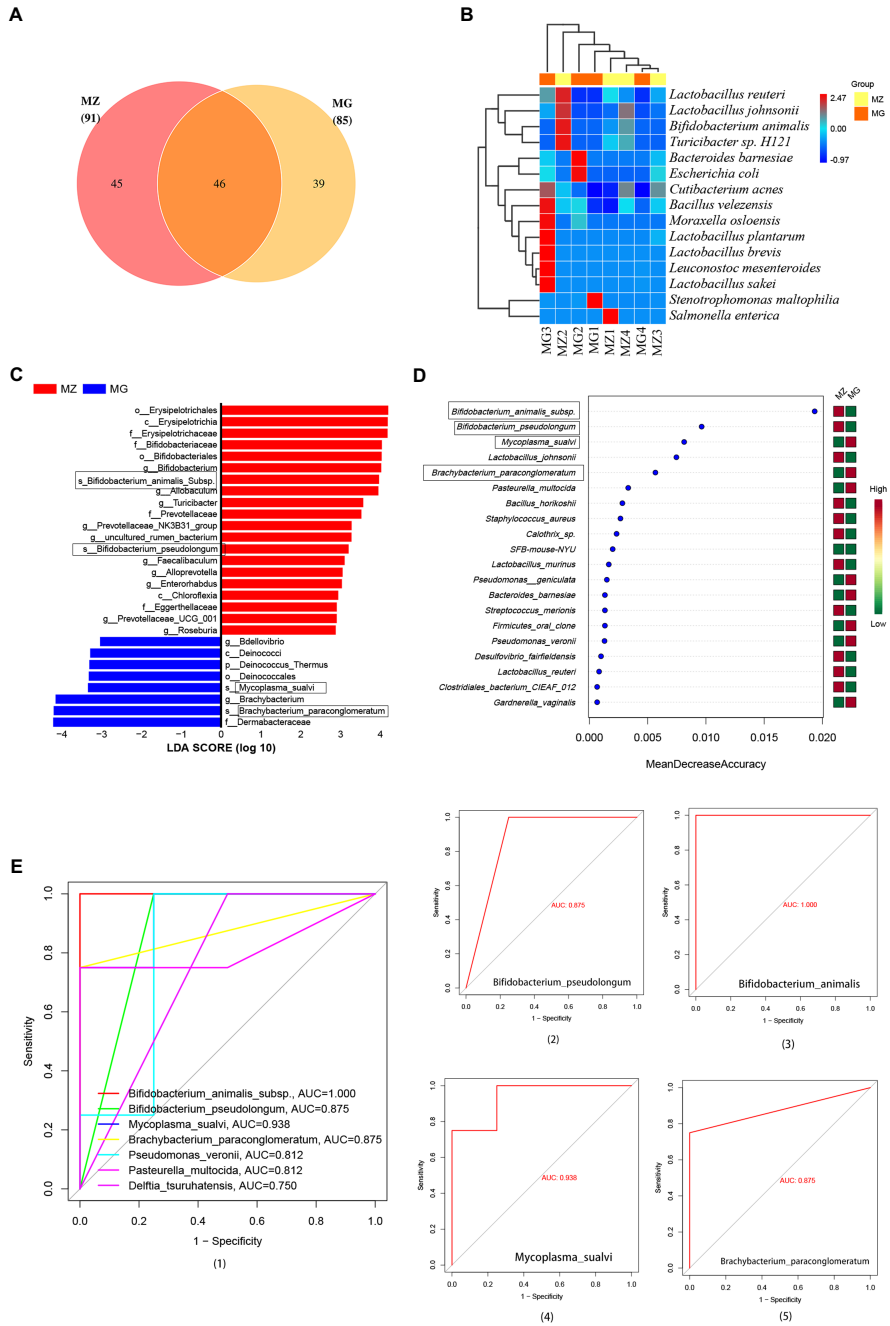
Intestinal mucosa microbiota biomarkers of hypertension induced by high salt diet. **(A)** OTU classification Venn diagram at species level. **(B)** Heatmap clustering of community species abundance at species level, and the closer the color is to red, the higher the abundance. **(C)** Cladogram generated from the LEfSe analysis indicating the phylogenetic distribution (LDA score ≥ 2, *p* < 0.05). **(D)** Random forest diagram of species level. **(E)** ROC curve of species level and ROC curve of intestinal mucosa microbiota biomarkers. MZ, normal group, MG, hypertensive group.

**Figure 6:**
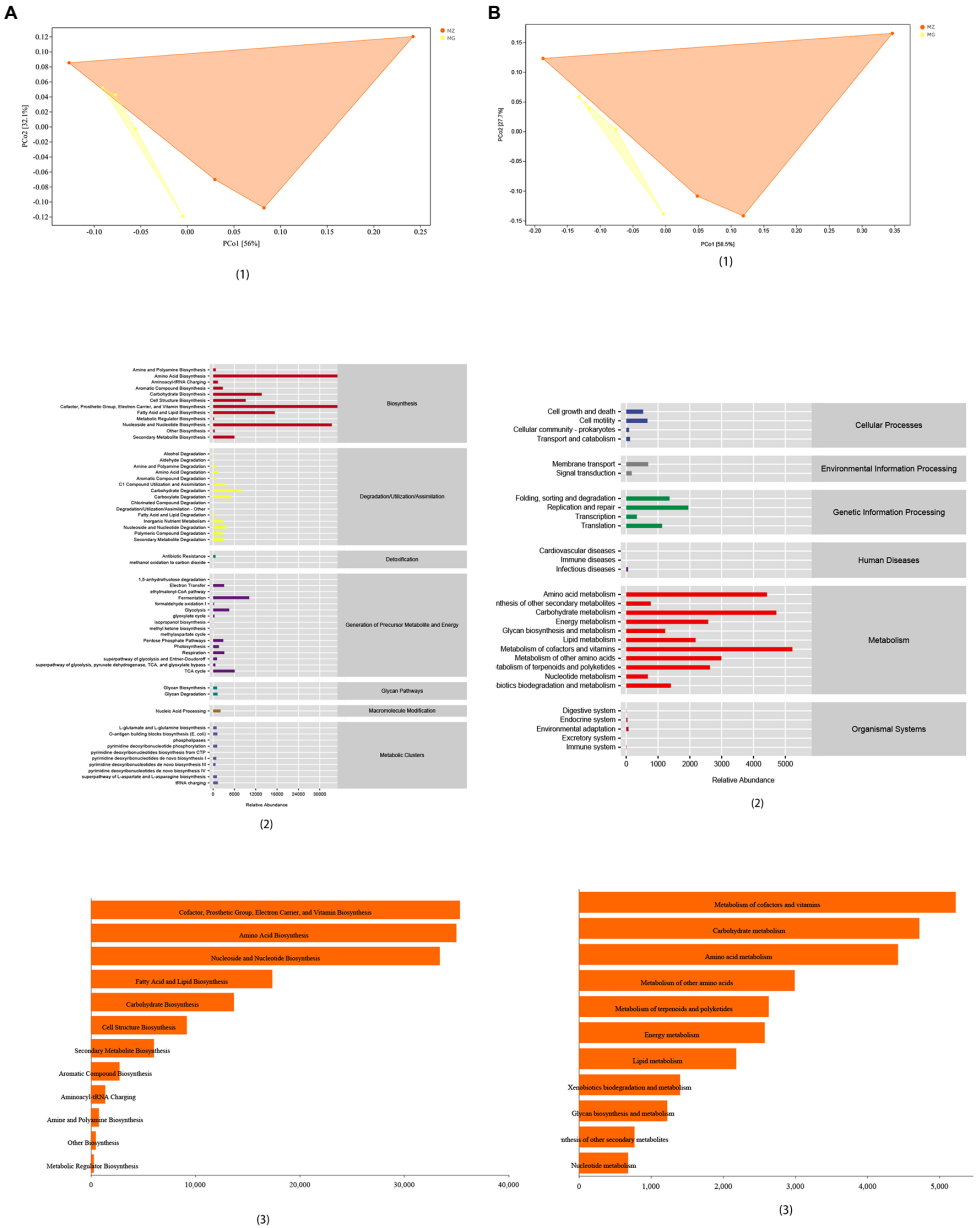
Functional analysis of intestinal mucosal microbiota. **(A)** PCoA map, predicted abundance and histogram of metabolic function with the greatest impact based on MetaCyc functional databases. **(B)** PCoA map, predicted abundance and histogram of metabolic function with the greatest impact based on KEGG functional databases.

#### 3.2.2. High salt diet leads to changes in cytokines and hormones associated with hypertension

Cytokines are the main arm of the immune system which are associated with hypertension. As can be seen from Figure 2B, the levels of IL-1β, IL-6, IL-8, and TNF-α in MG group were higher than MZ group, among which there were significant differences in IL-6, IL-8, and TNF-α between the two groups (*p* < 0.05), while there was no significant difference in IL-1β between the two groups (*p* > 0.05). Ang II, leptin and NE played a key role in the development of hypertension. Our research showed that the high salt diet resulted in an increase in the concentration of Ang II, LEP and NE in the serum of rats, and the concentration of Ang II in the MG group was significantly higher than that MZ group (*p* < 0.05) (Figure 2B2). The concentrations of LEP and NE were no significant difference between the two groups (*p* > 0.05) (Figures 2B6,7).

**Figure 7:**
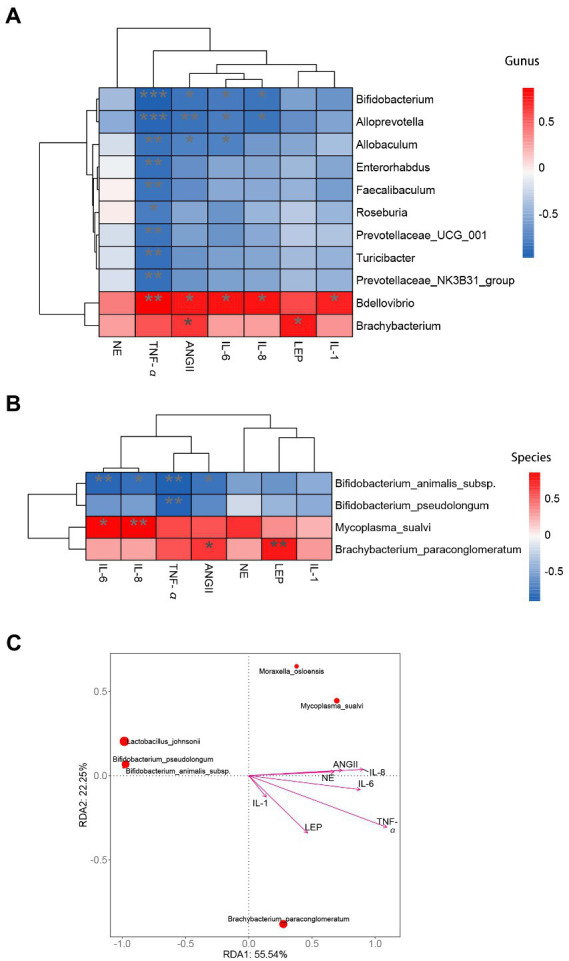
Correlation analysis of inflammatory cytokines and hormones with intestinal mucosa microbiota biomarkers associated with hypertension. **(A)** Heatmap analysis at genus level. **(B)** Heatmap analysis at species level. **(C)** RDA analysis at species level. *indicates significant correlation,** and *** indicate that the correlation is highly significant.

#### 3.2.3. High salt diet caused renal and vascular structure damage in rats

HE staining was performed on the kidneys and blood vessels of rats in MZ and MG groups. Figure 3A showed the complete and sectional views of the kidney of rats in the MZ and MG groups. Figure 3A1 showed the MZ group on the left and MG group on the right. In Figure 3A2, the first row is for the MZ group and the second row is for the MG group. From the perspective of kidney in the MZ and MG groups, the surface coat of kidney tissue was composed of connective tissue with uniform thickness. The renal parenchyma consists of superficial cortex and deep medulla, and the boundary between cortex and medulla is obvious. The number of cells and matrix in the glomeruli were uniform, which was evenly distributed in the cortex. However, there were also differences between MZ and MG kidneys. In the MZ group, renal tubular epithelial cell were round and plump, and the renal edges were arranged neatly and regularly. There was no obvious abnormalities in the medulla and no inflammatory changes in the kidney tissue (Figure 3A3). In the MG group, As can be seen from Figure 3B4, a small number of renal tubules and collecting ducts at the Cortical medulla junction were slightly dilated, with irregular lumen shape and flattened epithelium (black arrow). A high-salt diet caused vascular damage in rats. The HE staining result showed that the vascular structure of the MZ group was clear, the intimal endothelial cells were evenly distributed with normal morphology, the internal elastic plate was intact. The smooth muscle cells in the tunica media were arranged regularly with normal morphology and the connective tissue of the outer membrane was evenly distributed in rats of MZ group. There were no obvious inflammatory changes in the vascular tissue of normal rats (Figure 3B1). The hypertensive rats had local rupture of the vessel wall (black arrow), marked hemorrhage of the adventitia, visible red blood cells (red arrow), mild hyperplasia of local connective tissue, and fibrosis of a few cells (green arrow) (Figure 3B2).

### 3.3. High salt diet disturbs the microbiota of intestinal mucosa in rats

#### 3.3.1. The changes in the diversity and composition of intestinal mucosal microbiota

According to the Venn diagram, 781 OTUs were annotated in the MG group, 537 OTUs in the MZ group, and 182 OTUs were the same in the two groups (Figure 4A). We used multiple indicators of alpha diversity and beta diversity to analyze the inter-group and intra-group diversity of the MZ and MG groups (Figures 4B,C). From the α diversity, Chao1 and ACE indexes of MG group were higher than MZ group, and Shannon and Simpson indexes were lower than MZ group, but there were no significant differences in these four indexes between the two groups (Figures 4B1–4). In *β* diversity, PCoA1 contained 35.8% of the information, PCoA2 contained 25.26% of the information, and the stress of NMDS was 0.00982, indicating a remarkably separation of the two groups (Figures 4C1–4). According to the alpha and beta diversity, the richness and evenness of the intestinal mucosal microbiota of the two groups of rats were altered, and the species diversity between the groups was changed.

In addition to analyzing the changes of intestinal mucosal diversity in rats with high-salt diet, we also analyzed the top 15 dominant phylum, genus and species of mucosal microbiota (Figures 4D1,3,5). We also showed the dominant microbiota present only in the MZ or MG groups of phylum, genus, and species level (Figures 4D2,4,6). At the phylum level, Cyanobacteria, Proteobacteria and Firmicutes were the top three phyla in the MZ and MG groups, with abundances of 43.71 and 30.60%, 9.56 and 26.28%, 22.71 and 22.32%, respectively (Figure 4D1). Rokubacteria was only found in the MG group (Figure 4D2). At the genus level, among the top 15 dominant genera, the top three were *Stenotrophomonas*, *Lactobacillus* and *Bacillus*. There was no significant difference in these three genera between the two groups (Figure 4D3). The genus *Brachybacterium* was only present in group MG (Figure 4D4). At the species level, *Lactobacillus johnsonii* was the most abundant species in MZ group (2.42%), *Pseudomonas geniculata* was the most abundant species in MG group (17.80%) (Figure 4D5). Among the dominant species, *Mycoplasma sualvi* in the MG group was significantly higher than that in the MZ group (*p* < 0.05), *Brachybacterium paraconglomeratum* was only existed in the MG group, and *Bifidobacterium animalis* subsp., *Ornithinimicrobium* sp., *Desulfovibrio fairfieldensis*, *Bifidobacterium pseudolongum,* and *Delftia tsuruhatensis* were only existed in the MZ group (Figure 4D6). The results of relative abundance indicated that high-salt diet had a great impact on intestinal mucosal microbiota in rats.

#### 3.3.2. Potential biomarkers of intestinal mucosa in hypertensive rats induced by high-salt diet

At the species level, from the Venn diagram and the abundance heat map of each group, we could know that the MZ and MG groups contain 91 and 85 species respectively, and the two groups have 46 identical species, 45 species are unique to the MZ group and 39 species are unique to the MG group (Figure 5A). The MZ group mainly contains *Bifidobacterium animalis* subsp., *Lactobacillus reuteri*, *Turicibacter* sp. and *Lactobacillus johnsonii*, while the MG group mainly contains *Moraxella osloensis*, *Stenotrophomonas maltophilia,* and *Leuconostoc mesenteroides* (Figure 5B). In our study, we used LEfSe to screen for bacteria that could significantly distinguish intestinal mucosal microbiota between normal diet rats and high-salt diet rats, which were potential biomarkers of hypertension induced by high-salt diet. LDA score (log 10) greater than 2 was used as the evaluation criterion for LEfSe analysis and there are 28 signature bacterial taxa in LEfSe analysis (4 species, 12 genera, 5 families, and 3 orders) which can be used as mucosal microbial markers of the rat in response to high salt diet (Figure 5C). As can be seen, at the species level, the microbial markers were *Bifidobacterium animalis* subsp., *Bifidobacterium pseudolongum*, *Mycoplasma sualvi*, *Brachybacterium paraconglomeratum* (Figure 5C).

In addition to LEfSe analysis, we also constructed a random forest diagnostic model at the species level to judge the importance of species to the two groups, and the species with the highest importance in the random forest maybe the marker species for differences between groups. As can be seen from the random forest at the species level, from the perspective of influence value, the top five bacteria were *Bifidobacterium animalis* subsp.*, Bifidobacterium pseudolongum, Mycoplasma sualvi, Lactobacillus johnsonii,* and *Brachybacterium paraconglomeratum* (Figure 5D). Based on random forest analysis, we performed ROC analysis on these bacteria and the result showed that there were six species with AUC values greater than 0.8 (Figure 5E1), which are *Bifidobacterium animalis* subsp. (AUC = 1.000), *Bifidobacterium pseudolongum* (AUC = 0.875), *Mycoplasma sualvi* (AUC = 0.938), *Brachybacterium paraconglomeratum* (AUC = 0.875), *Pseudomonas veronii* (AUC = 0.812), and *Pasteurella multocida* (AUC = 0.812) (Figures 5E2–5). According to LEfSe analysis and random forest analysis, *Bifidobacterium animalis* subsp., *Bifidobacterium pseudolongum*, *Mycoplasma sualvi,* and *Brachybacterium paraconglomeratum* were appeared in both analyses. We analyzed the combination of the significantly different species with the top 15 dominant species, *Bifidobacterium animalis* subsp., *Mycoplasma sualvi,* and *Brachybacterium paraconglomeratum* were the dominant differential bacteria. *Bifidobacterium animalis* subsp. only exists in MZ group (0%: 2.521%) *and Brachybacterium paraconglomeratum* only exists in MG group (2.419%: 0%). The results indicated that *Bifidobacterium animalis* subsp. and *Brachybacterium paraconglomeratum* were all belong to the Actinobacteria phylum and Actinobacteria Class, which can be used as a potential biomarker of the intestinal mucosal microbiota in response to high salt diet.

### 3.4. High-salt dietary changes the function of intestinal mucosal microbiota in rats

We used phylogenetic investigation of communities by reconstruction of unobserved states (PICRUSt2) to predict the sample function. We predicted the microbial 16S rRNA gene sequences obtained in this study in MetaCyc and KEGG functional databases. The samples showed significant separation analyzed by the two databases (Figures 6A1,B1). Based on the KEGG functional databases, it could be known that the changes of intestinal mucosal microbiota caused by high-salt diet mainly affect the metabolic function of rats, which mainly affected metabolism of vitamin and cofactors, amino acid metabolism and carbohydrate metabolism (Figure 6A2). According to MetaCyc database, high-salt diet mainly affected the microbiota related to biosynthesis in rat mucosa, which mainly affected the amino acid biosynthesis, prosthetic group, cofactor, electron carrier, vitamin biosynthesis, and nucleoside and nucleotide biosynthesis (Figure 6B2). Combined these two databases, the functional prediction showed that high-salt diet mainly affected the microbiota related to the biosynthesis and metabolism of vitamin, amino acid and cofactor in the intestinal mucosa of rats (Figures 6A3,B3).

### 3.5. High salt diet affects the interaction between differential bacteria of intestinal mucosa and blood indexes related to hypertension in rats

Based on the result of blood markers associated with hypertension and intestinal mucosal differential bacteria we performed correlation analyses by Spearman correlation coefficients and RDA analysis. In terms of the genus level, the differential bacteria *Bifidobacterium* have a significant correlation with the IL-6, IL-8, TNF-α, and AngII (Figure 7A). *Brachybacterium* had significant effect on AngII (Figure 7A). At the species level, *Bifidobacterium animalis* subsp. had a significant correlation with TNF-α, IL-6, AngII and IL-8, *Brachybacterium paraconglomeratum* had a significant correlation with Ang II, and *Mycoplasma_sualvi* was correlated with IL-8 (Figures 7B,C). Therefore, we can infer that *Bifidobacterium animalis* subsp. was the mucosal microbial marker of hypertension caused by high salt intake and promoted inflammation in the body.

## 4. Discussion

As more and more prepackaged foods had entered people’s lives, people consumed large amounts of sodium ions through their daily diet and prepackaged foods, and a high-salt diet had become the norm. Hypertension is one of the major hazards of a high-salt diet, and excessive elevation of blood pressure caused by salt load was the characteristic of salt-sensitive hypertension (Lee et al., 2020). Studies have shown that inflammation and immunity play an important role in the development and progression of hypertension. Innate and adaptive immune cells entered blood vessels and kidneys and released matrix metalloproteinases, cytokines and other mediators, which leaded to tissue damage and dysfunction (Xiao and Harrison, 2020). In this study, the inflammatory and immune responses to high salt diet-induced hypertension and end-organ damage were investigated, which correlated the microbiota on intestinal mucosa (immune organ) and blood indicators related to hypertension, so as to systematically answered the role of inflammation and immunity in the formation of hypertension induced by high-salt diet.

From the results of our study, high salt diet could raise blood pressure and lead to hypertension. High salt diet significantly increased the food intake and urine output of the rats, but the body weight of the rats was significantly lower than that of the normal rats. This may be because the metabolism of rats in the MG group is faster than that in the MZ group and we have two results to support this theory. Firstly, the activities of several intestinal digestive enzymes measured by us in the MG group are higher than those in the MZ group (data not shown in this article). Secondly, we observed that the tail lifting of rats in the MG group is irritable, restless, and the amount of exercise was much higher than that of MZ group. These results suggested that high salt-induced hypertension may have increased food intake while losing body weight. Our results also support the theory that a high-salt diet induced hypertension without causing changes in the blood lipid (HDL-C, LDL-C, TG, and CHOL) parameters.

Diet has been one of the most extensively factors affecting the microbial composition, which can indirectly induce inflammatory diseases. Inflammatory response was a process of self-limiting and self-protection, but if self-limiting process was not properly regulated, it can lead to chronic and harmful inflammation, and chronic low-grade inflammation could lead to high blood pressure (Mehaffey and Majid, 2017; FElijovich et al., 2020). Proinflammatory cytokines played a key role in inflammation and tissue damage (Sepehri et al., 2016). Serum levels of proinflammatory cytokines were elevated in hypertensive patients. Ang II could damage endothelial cells and enhance sympathetic nerve stimulation, so as to increase blood pressure (Lv et al., 2021). This study comprehensively evaluated the changes of inflammation caused by high-salt diet in rats from the aspects of inflammatory cytokines (TNF-α, IL-8, IL-1β, and IL-6) and hormones related to hypertension (NE, LEP, and Ang II), and Vascular and renal lesions were also examined. The results of our study showed that major proinflammatory factors TNF-α, IL-8, and IL-6 in the blood of rats in MG group were significantly increased, suggesting that high-salt diet could lead to the increase of proinflammatory factors. Among the hormones related to hypertension, Ang II was significantly increased in MG group. We hypothesized that a high-salt diet may increase serum levels of IL-6, IL-8, TNF-α, and Ang II to promote hypertension. HE staining of the kidney and vessels showed that the renal tubules and collecting ducts at the junction of the cortical layer in the kidney of hypertensive rats were slightly dilated, the vascular wall was partially broken, and red Erythrocytes were seen in the outer membrane. Based on the above we can speculate that high-salt diet leads to significant change in inflammatory cytokines and Ang II, which may lead to continuous low-grade inflammation in the body, promote the formation of hypertension, and promote end-organ damage, so as to further aggravates inflammation in the body and promotes the continuous rise of blood pressure.

Human immune organs include thymus, bone marrow, lymph nodes, spleen and digestive tract mucosa. Host mucosal immunology supported a variety of potential pathways for regulating inflammatory responses and could be used to reveal complex diet–microbe–host interactions and interdependence (Bron et al., 2011). As we all known, the spleen is the largest peripheral immune organ and the thymus is an important lymphoid organ in the body. The spleen has functions of blood storage, hematopoiesis, removal of senescent red blood cells and immune response (Lewis et al., 2019; Ji et al., 2020). Immune organ index and intestinal mucosal microbiota were selected as our research objects for the immune coefficient of high salt diet-induced blood pressure increase in rats. We measured liver coefficient, thymus coefficient and spleen coefficient in rats with high salt induced hypertension, and the results showed that spleen coefficient in MG group was significantly higher than MZ group and thymus coefficient and liver coefficient in MG group were slightly higher than those in MZ group without significant difference, which suggested that high salt diet can cause changes in the immune organ spleen during the process of hypertension in rats. In terms of intestinal mucosal microbiota, alpha and beta diversity indicate that samples differ little within the group and can be clearly distinguished between MZ and MG groups and high-salt diet could interfere with the composition of intestinal mucosal microbiota in rats. From the intestinal mucosa to the luminal and then to the fecal side, the gut microbiota was constantly changing. Study had shown that the colonization of gut microbiota in the intestinal mucosa is more stable and sensitively to reflect the microbiota ectopic colonization, which is most closely related to host immunity (Schütte et al., 2021; Gu et al., 2022). From phylum level to species level, we performed a taxonomic level of relative abundance analysis. From the phylum level, we found a interesting phenomenon that Cyanobacteria is the most abundant phylum, followed by Proteobacteria and Firmicutes. This is quite different from the usual knowledge that Firmicutes and Bacteroidetes are the major phylum in intestinal feces and it also further confirmed the theory of different distribution of microbiota in intestinal mucosal, intestinal lumen and stool parts. From the species level, the research indicated *Bifidobacterium Animalis* subsp. and *Brachybacterium paraconglomeratum* were the key symbiont dominant bacteria of intestinal mucosa with high salt diet intake. Association analysis shown that *Bifidobacterium animalis* subsp. was only existed in MZ group and it was significantly correlated with the changes of IL-6, IL-8, TNF-α and Ang II. Therefore, we can speculate that *Bifidobacterium animalis* subsp. was the intestinal mucosal characteristic species of hypertension induced by high salt diet. *Bifidobacterium animalis* subsp. was probiotic that help relieve diseases associated with intestinal peristalsis disorders (Cheng et al., 2021) and it also could significantly relieved constipation and improved immune function (Lee et al., 2017; Wang et al., 2021). Combined *Bifidobacterium animalis* subsp. *lactis* with other probiotics showed anti-inflammatory activity, and it might be novel adjuvants for inflammatory bowel disease therapy (Li et al., 2019). It may be a potential candidate in combating obesity (Uusitupa et al., 2020) and could reduce proinflammatory factor secretion (IL-8), enhance intestinal barrier function (Xu et al., 2022). Based on the above, we could draw the conclusion that high salt diet mediated chronic low-grade inflammation through inhibited the growth of *Bifidobacterium animalis* subsp. in intestinal mucosa and caused end-organ damage, which leads to the increase of blood pressure. However, due to the current intestinal mucosal microbiota research is limited. Further investigation of *Bifidobacterium animalis* subsp. as a possible adjuvant for the treatment of hypertension induced by high salt intake can be conducted and the physiological effects of intestinal mucosal microbiota on the association between diet and body diseases also need to further investigate. We believe that this study provides a new perspective on inflammation and immunity to explain the relationship between high-salt diet interference with intestinal mucosal microbiota and body diseases.

## Data availability statement

The datasets presented in this study can be found in online repositories. The names of the repository/repositories and accession number(s) can be found at: https://www.ncbi.nlm.nih.gov/, PRJNA910332.

## Ethics statement

The animal study was reviewed and approved by Animal Ethics and Welfare Committee of Hunan University of Chinese Medicine.

## Author contributions

TZ designed the study, performed the experimental work, analyzed the data, and wrote the manuscript. YW contributed to animal feeding and hypertension measurement. K-xG performed the experimental work and analyzed the data. Z-jT and TY designed the study. All authors revised the manuscript and approved the final version.

## Conflict of interest

The authors declare that the research was conducted in the absence of any commercial or financial relationships that could be construed as a potential conflict of interest.

The reviewer BL declared a shared affiliation with the authors YW and Z-jT to the handling editor at the time of review.

## Publisher’s note

All claims expressed in this article are solely those of the authors and do not necessarily represent those of their affiliated organizations, or those of the publisher, the editors and the reviewers. Any product that may be evaluated in this article, or claim that may be made by its manufacturer, is not guaranteed or endorsed by the publisher.

## Funding

This study was financially supported by innovation and entrepreneurship training program for college students of Hunan Province, China (S202010541049), Innovation and entrepreneurship training program for college students of Hunan University of traditional Chinese medicine (X201910541035).
